# Global Methylomic and Transcriptomic Analyses Reveal the Broad Participation of DNA Methylation in Daily Gene Expression Regulation of *Populus trichocarpa*

**DOI:** 10.3389/fpls.2019.00243

**Published:** 2019-02-28

**Authors:** Lixiong Liang, Yingying Chang, Junqian Lu, Xiaojuan Wu, Qi Liu, Weixi Zhang, Xiaohua Su, Bingyu Zhang

**Affiliations:** ^1^State Key Laboratory of Tree Genetics and Breeding, Research Institute of Forestry, Chinese Academy of Forestry, Beijing, China; ^2^Key Laboratory of Tree Breeding and Cultivation of State Forestry Administration, Research Institute of Forestry, Chinese Academy of Forestry, Beijing, China

**Keywords:** *Populus* L., daily gene expression, DNA methylation, DMRs, circadian clock

## Abstract

Changes in DNA methylation patterns in different tissues, at various developmental stages, and under environmental stimuli have been investigated in plants. However, the involvement of DNA methylation in daily gene expression regulation and the plant circadian clock have not been reported. Here, we investigated DNA methylomes and mRNA transcriptomes from leaves of *P. trichocarpa* over 24 h by high-throughput sequencing. We found that approximately 15.63–19.50% of the genomic cytosine positions were methylated in mature poplar leaves, with approximately half being in the form of asymmetric CHH sites. Repetitive sequences and transposable elements (TEs) were heavily methylated, and the hAT and CMC-EnSpm transposons were more heavily methylated than other TEs. High methylation levels were observed upstream and downstream of the transcribed region, medium in exon and intron, low in untranslated region (5′-UTR and 3′-UTR) of genic regions. In total, about 53,689 differentially methylated regions (DMRs) were identified and CHH context was the most abundant type among daily DNA methylation changes. The DMRs overlapped with over one third of the total poplar genes, including plant defense genes. In addition, a positive correlation between expression levels and DNA methylation levels in the gene body region were observed in DMR overlapping genes. About 1,895 circadian regulated genes overlapped with DMRs, with 871 hypermethylated genes with down-regulated expression levels and 881 hypomethylated genes with up-regulated expression levels, indicating the possible regulation of DNA methylation on the daily rhythmic expression of these genes. But rhythmic DNA methylation changes were not detected in any oscillator component genes controlling the plant circadian clock. Our results suggest that DNA methylation participates widely in daily gene expression regulation, but is not the main mechanism modulating the plant circadian clock.

## Introduction

DNA methylation is an epigenetic modification that plays an important role in plant development and environmental adaption (Gehring, 2013; Bilichak and Kovalchuk, 2016). In plant genomes, DNA methylation occurs in all sequence contexts, including symmetric CG and CHG sites and non-symmetric CHH sites (in which H = A, T, or C) (Law and Jacobsen, 2010). In *Arabidopsis*, methylation rates for CG, CHG, and CHH are approximately 24.0, 6.7, and 1.7%, respectively (Cokus et al., 2008). While in *P. trichocarpa*, they are approximately 41.9, 20.9, and 3.25%, respectively (Feng et al., 2010). The mechanisms responsible for the establishment and maintenance of plant DNA methylation have been clarified for *Arabidopsis*. *de novo* DNA methylation is mediated by RNA-directed DNA methylation pathways established by DOMAINS REARRANGED METHYLTRANSFERASE 2 (DRM2), while maintenance of DNA methylation in CG and CHG contexts is catalyzed by DNA METHYLTRANSFERASE 1 (MET1) and CHROMOMETHYLASE 3 (CMT3), respectively, and maintenance of DNA methylation in CHH is carried out by DRM2 and CHROMOMETHYLASE 2 (CMT2) (Law and Jacobsen, 2010; Stroud et al., 2014; Zhang et al., 2018). Active demethylation was also reported in plants, indicating that DNA methylation is dynamic rather than static (Agius et al., 2006; Penterman et al., 2007; Zhu, 2009; Kim and Zilberman, 2014). Due to these dynamic characteristics, changes in genomic DNA methylation patterns have been reported in plant stress responses, environmental adaption, and different developmental stages (Raj et al., 2011; Yaish et al., 2011; Dowen et al., 2012; Vining et al., 2012).

Circadian rhythms produce a biological measure of time and regulate many biological processes in diverse life forms (Bell-Pedersen et al., 2005; Wijnen and Young, 2006; Atkins and Dodd, 2014). In mammals, the cell-autonomous molecular clock is generated by two interlocking transcription/translation feedback loops (TTFLs) that act in concert to produce robust 24-h rhythms of gene expression (Partch et al., 2014). In plants, the circadian system consists of three interconnected positive–negative transcriptional feedback loops called the central, morning, and evening loops that lead to time-specific expression of clock components, such as LATE AND ELONGATED HYPOCOTYL (LHY), CIRCADIAN AND CLOCK ASSOCIATED 1 (CCA1), and TIME OF CAB EXPRESSION (TOC1) (Harmer, 2009; Pruneda-Paz et al., 2009; Herrero and Davis, 2012). Recent studies have shown that epigenetic mechanisms regulate the circadian clock in *Neurospora*, mice, and humans (Azzi et al., 2014; Cedernaes et al., 2015; Cronin et al., 2017). In the human brain, DNA methylation cycles modulate the rhythmic expression of clock genes (Cronin et al., 2017); a single night of wakefulness can alter the DNA methylation status of core clock genes (*BMAL1*, *CLOCK*, *CRY1*, and *PER1*) and subsequently alter their transcriptional profiles in key metabolic tissues (Cedernaes et al., 2015). In mice, the suprachiasmatic nucleus of the hypothalamus (the master clock tissue in mammals) utilizes DNA methylation as a mechanism to drive circadian clock plasticity (Azzi et al., 2014). To date, only a few studies have been conducted on the epigenetic regulation of clock genes in plants. For example, *Arabidopsis* circadian clocks utilize the histone demethylase JMJD5 to demethylate H3K36, and rhythmic transcription of Arabidopsis clock genes is regulated by rhythmic histone modifications (Jones et al., 2010; Lu et al., 2011; Malapeira et al., 2012; Song and Noh, 2012). A study of hybrid vigor in *Arabidopsis* showed that increased genome-wide DNA methylation in F1 hybrids may lead to the downregulation of two circadian oscillator genes (CCA and LHY), thereby altering the circadian rhythm (Shen et al., 2012). This finding suggests that DNA methylation might also regulate the plant circadian clock, as in *Neurospora* and mammals. However, to date, the involvement of DNA methylation in daily gene expression regulation and the plant circadian clock has not been fully explored.

As a model forest tree taxon, poplar (*Populus* L.) can reproduce asexually to rapidly produce genetically identical clones, which serve ideal plants for the study of daily variations in processes such as DNA methylation and gene expression. In this study, we used a sequenced clone of *P. trichocarpa*, Nisqually-1, to investigate daily changes in DNA methylomes by whole-genome bisulfite sequencing (BS-seq), as well as mRNA transcriptome by RNA sequencing (RNA-seq). We generated a detailed methylome of mature *P. trichocarpa* leaves, and found that CHH context was the most abundant type of daily DNA methylation change. We also noted widespread involvement of DNA methylation regulation in daily gene expression, and found an overall positive correlation between methylation level and gene expression level. Although the expression of some circadian-regulated genes and a circadian clock gene might be regulated by DNA methylation, this was not the main mechanism driving the plasticity of the circadian clock in *Populus*.

## Results

### DNA Methylome of *P. trichocarpa* Leaves

The methylomes of mature *P. trichocarpa* leaves were collected at nine time points and measured by BS-seq (Supplementary Figures S1, S2). Each methylome was sequenced to over 30-fold coverage, and the conversion efficiency of the sodium bisulfite reaction was over 99% (Supplementary Table S1). Approximately 66.17–71.05% of reads were uniquely mapped to the reference for each library, with 5× cytosine coverage over 93% (Supplementary Table S2).

In the nine samples assayed, approximately 15.63–19.50% of the genomic cytosine positions were methylated (Supplementary Table S3), with approximately half in the form of asymmetric CHH sites (Supplementary Figure S3). This result was similar to that for tomato fruit, but in contrast to that for *Arabidopsis* leaves, in which more than half of the cytosine occurred in the form of symmetric CG sites (Zhong et al., 2013).

In the CG, CHG, and CHH sites, nearly one half (42.53–46.66%) of the CG sites, one third (28.51–31.98%) of the CHG sites, and one tenth (10.07–13.00%) of the CHH sites were methylated (Supplementary Table S3). The average methylation levels at CG, CHG, and CHH sites were 43.99, 29.84, and 11.57%, respectively; these levels were higher than those reported in a previous study of *P. trichocarpa* leaves (41.9, 20.9, and 3.25%, respectively), especially at the CHG and CHH sites (Feng et al., 2010). In that study, more than one third of the genomic sequence in scaffolds, which was enriched in repetitive DNA, was excluded from the analysis. This may be the main reason for the difference between studies. We suspect that CHG and CHH methylation sites with higher methylation levels may occupy a larger proportion of the repetitive regions of the poplar genome than those of CG methylation sites.

**FIGURE 1:**
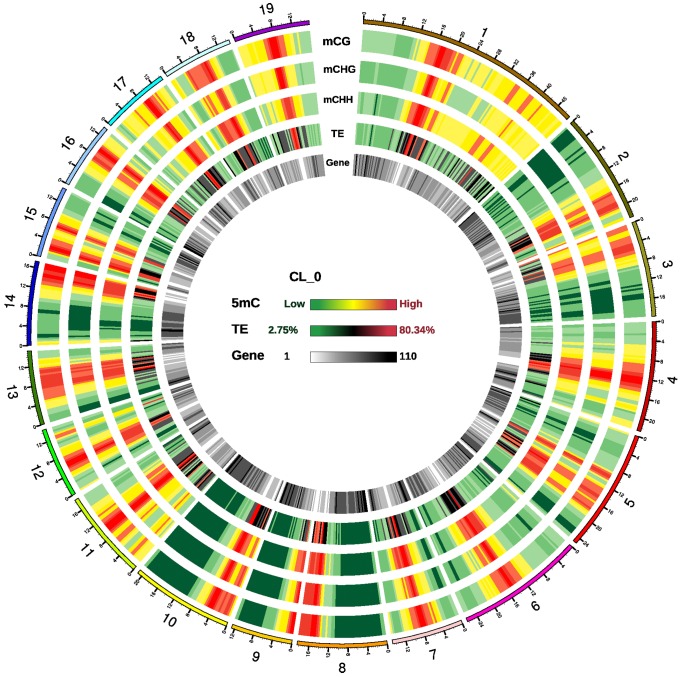
Epigenomic analysis in *P. trichocarpa* leaves. Density plot of 5-methylcytosine in three sequence contexts [mCG, mCHG, and mCHH (where H = A, C, or T), transposable elements (TEs), and genes in sample CL_0]. High density regions of cytosine methylation were distributed in all 19 chromosomes, which were enriched in TEs but sparse in genes. Chromosome numbers and scales are indicated on the outer rim. Chromosome distribution of 5-methylcytosine in all samples are similar, and not present in here.

Regions with a high density of cytosine methylation were observed in all 19 chromosomes, which were enriched in transposable elements (TEs) but sparse in genes (Figure 1 and Supplementary Figure S4). In these methylation-rich regions, all three contexts (CG, CHG, and CHH) had high methylation density. In genic regions, the methylation density of all three contexts was much higher in promoter regions than in exons, introns, 3′UTRs (untranslated regions) or 5′UTRs (Supplementary Figure S5).

The average methylation level in each chromosome differed, with the lowest in chromosome 9 and the highest in chromosome 17 (Supplementary Figure S6). The methylation levels at CG, CHG and CHH sites were consistent with a previous study of *P. trichocarpa* leaves, with the highest levels at CG, medium levels at CHG, and the lowest levels at CHH sites (Feng et al., 2010; Supplementary Figure S7). With regard to the methylation levels in different genic regions, methylation levels were high in upstream and downstream of the transcribed region, medium in exon and intron, low in untranslated region (5′-UTR and 3′-UTR) (Supplementary Figure S8). High levels of methylation were seen in all TE types, and the hAT and CMC-EnSpm transposons tended to be more heavily methylated than other TEs, suggesting that control of these two types of transposons may be important in poplars (Supplementary Figure S9).

Using the traditional BS-seq method, we picked 294 bp exon region of gene POPTR_0012s08770s (chr12_9852031-9852327) to validate methylation level in three randomly selected samples (CL0, CL4, and CL8), and 269 bp promoter region of gene POPTR_0015s02230 (chr15_1573578-1573846) in three randomly selected samples (LD4, CL0, and CL8), and the results were generally consistent with those from our whole genome BS-Seq analyses (Supplementary Figures S10, S11), indicating the reliability of our BS-Seq results.

### Daily DNA Methylomic Changes in *P. trichocarpa* Potentially Influenced Gene Transcription in Multiple Biological Processes

To detect daily DNA methylation changes in the poplar genome, we used a sliding window approach to screen for differentially methylated regions (DMRs) between four pairs of five groups (G2 vs. G1, G3 vs. G2, G4 vs. G3, and G5 vs. G4). The circadian treatment and sample grouping in the data analysis were described in details in Methods (Supplementary Figure S1). A total of 53,689 DMRs were identified, representing ∼3% of the poplar genome (Supplementary Figure S12). The maximum length of the DMRs was 1934 bp, and the largest number of methylated cytosines in each DMR was 307. The overall ratio of hypo- to hypermethylated DMRs was 1.15, with group G3 vs. G2 having 2.76-fold more hypo DMRs than hyper DMRs, indicating that more genes were demethylated to meet the requirements of life activities during this period (Supplementary Table S4). In addition, approximately 52.73% of DMRs were located in genic regions, with 70.59% in promoter regions, followed by exons (14.19%), introns (13.91%), and UTRs (1.31%) (Supplementary Figure S13). A total of 2,134,101 of the cytosine positions were identified as differentially methylated cytosines (DMCs), and the CG, CHG, and CHH contexts of the DMCs differed, with the highest proportion being located in CHH (63.84%), followed by CHG (28.18%), and the lowest being in CG (7.98%) (Supplementary Figure S14). Thus, daily DNA methylation changes occurred mainly on CHH sites. Approximately 12.90% of the DMCs were located in genic regions, with 70.59% in promoters, 14.19% in exons, 13.91% in introns, and 1.31% in UTRs (Supplementary Figure S15).

**FIGURE 2:**
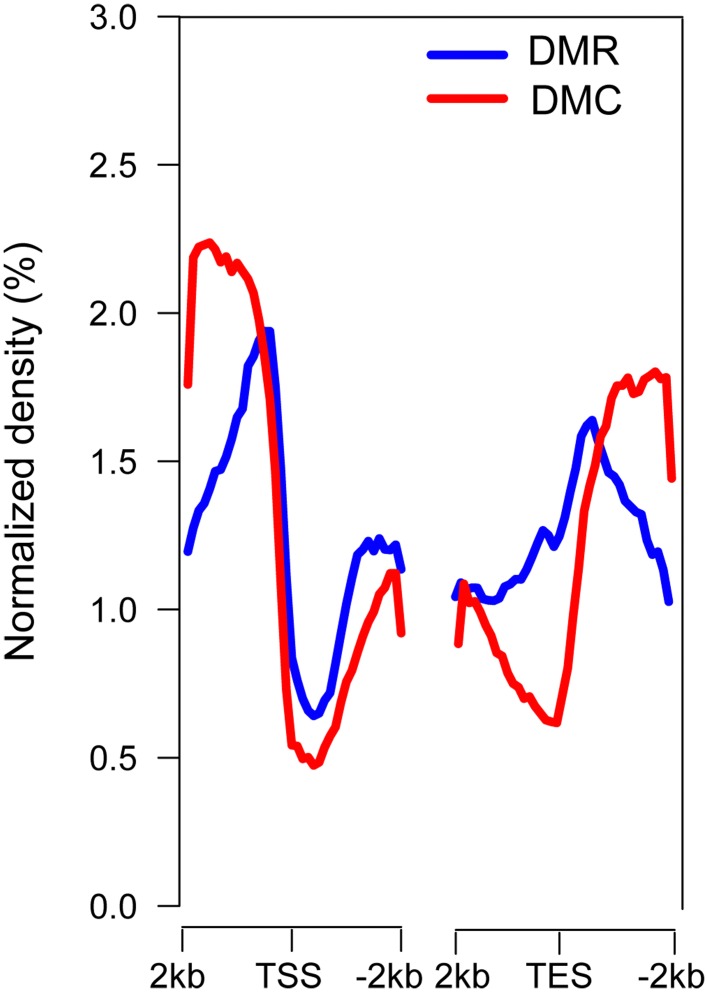
Distribution of DMRs and DMCs in genic regions. TSS, transcriptional start site; TES, transcriptional end site. TSS and TES exhibited the lowest densities of DMRs and DMCs, and the 5′ upstream and 3′ downstream gene regions exhibited higher densities of DMRs and DMCs. The 2 kb regions were divided into 20 bins, and the average methylation levels were calculated.

An uneven distribution of DMRs and DMCs was observed in all 19 chromosomes (Supplementary Figure S16). In genic regions, higher densities of DMRs and DMCs were found in both the 5′ upstream and 3′ downstream regions of genes and in the region between the transcriptional start site (TSS) and transcriptional end site (TES) (Figure 2), indicating potential regulatory regions for DNA methylation involved in gene expression in poplar.

About 4,832–7,564 genes overlapped with DMRs, and these genes may be potentially affected by DMRs in the four comparisons (Supplementary Figure S17). In total, 15,300 genes overlapped with DMRs, nearly one third of the total poplar genes, suggesting the broad involvement of DNA methylation in daily gene expression. The DMR overlapping genes were annotated with up to 4,270–4,925 GO terms, 61.83% of which were biological process terms, 10.27% cellular component terms, and 27.91% molecular function terms. We performed GO enrichment of the DMR overlapped genes in each comparison and identified significantly enriched GO terms (FDA < 0.05). Specific significantly enriched GO terms were found in three pairs of groups (G2 vs. G1, G4 vs. G3, and G5 vs. G3) (Supplementary Table S5). The specific significantly enriched GO terms in G4 vs. G3 mostly involved biological processes, such as protein modification, phosphate metabolism, and macromolecule metabolism, indicating the involvement of DNA methylation in active physiological processes in the subjective morning, such as photosynthesis and transpiration. The specific significantly enriched GO terms in G5 vs. G4 were mainly related to defense response and signal transduction (e.g., programmed cell death, immune system processes, and receptor activity). In Arabidopsis, defense genes are regulated by the circadian clock; Col-0 plants display the greatest resistance to the virulent bacterial pathogen Pst DC3000 in the morning, and greatest susceptibility in the evening (Bhardwaj et al., 2011). We therefore hypothesized that plant defense gene expression was mainly regulated by the circadian clock through DNA methylation. KEGG analysis showed that the DMR overlapping genes revealed in the four comparisons are involved in 119–122 pathways, including RNA transport, fatty acid metabolism, carbon fixation in photosynthetic organisms, plant–pathogen interaction, and biosynthesis of amino acids, but no significant pathway was found (Supplementary Figure S18). In summary, we found that the DMR overlapped genes were involved in multiple biological processes and diverse pathways, indicating the importance of DNA methylation in poplar cells.

### Correlation Between DNA Methylation Level and Daily Gene Expression in *P. trichocarpa*

**FIGURE 3:**
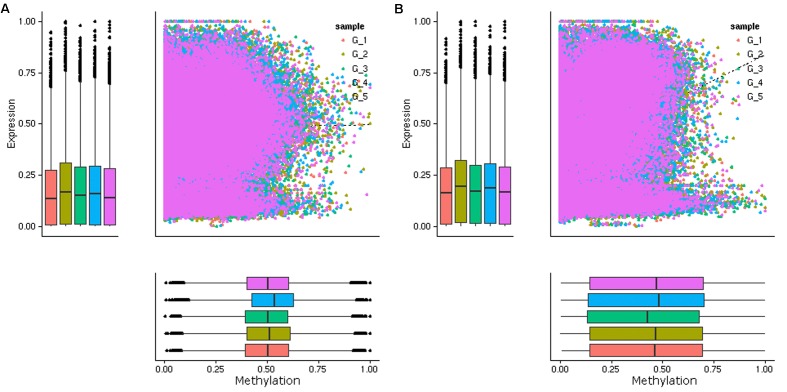
Scatterplot of DNA methylation (*x*-axis) and gene expression (*y*-axis) of the DMRs overlapped genes in five groups (G1, G2, G3, G4, and G5). The genes with DMRs in the promoter regions **(A)** and the gene body regions **(B)** were presented. A overall positive correlation was observed between the methylation levels in the gene body and the mRNA expression levels of these DMRs overlapped genes, while a overall negative correlation between the methylation levels in the promoter regions and the mRNA expression levels was not observed.

Previous studies have shown that DNA methylation levels in genic regions affect gene expression, with promoter methylation associated with repression of gene expression and CG gene body methylation associated with intermediate expression of genes (Zhang et al., 2006; Takuno and Gaut, 2012; Wang et al., 2015). Given the intrinsic relationship between DNA methylation and gene expression, we performed a transcriptomic analysis of leaf samples collected at each time point by mRNA sequencing (Supplementary Table S6). The correlations between DNA methylation and the expression levels of genes were analyzed. In contrast to our expectation, an overall negative correlation between the methylation level in the promoter regions (2.0 kb upstream the TSS) and the mRNA expression level was not observed (Figure 3A). Then we examined the correlations between DNA methylation level in shorter regions upstream the TSS (0.5, 1.0, and 1.5 kb) and downstream the TTS, very weak negative correlation was only found in 0.5 kb downstream the TTS (Supplementary Figures S19, S20). However, a negative correlation between the methylation level in the promoter region and the mRNA expression level of that gene was observed for certain genes (Supplementary Figure S21). Previous research has indicated that C methylation in gene body (i.e., between start and stop codons) is associated with the efficiency and accuracy of transcription and is positively correlated with gene expression (Li et al., 2012; Xu et al., 2018). Consistent with the previous results, a positive correlation between C methylation in gene body and the mRNA expression levels of genes was observed (Figure 3B). These results indicated that C methylation occurring in the body of codding genes played a more important role in the daily regulation of gene expression in poplar.

Six genes were randomly selected from the DMR overlapped genes, and their expression and C methylation levels in their promoter region and gene body region were obtained from the transcriptome and methylome data, respectively. The expression of the six genes was further validated by quantitative real-time polymerase chain reaction (qPCR). The results were generally consistent with those of the RNA-seq analysis (Supplementary Figure S22); however, a negative correlation between expression and methylation levels in the promoter, as well as a positive correlation between expression and methylation levels in the gene body region of the detected genes, were observed only in some groups. For example, the expression level of POPTR_0006s06900 was negatively correlated with the methylation level in both the promoter and the gene body region (Supplementary Figure S23). Therefore, the relationship between gene expression and methylation is an overall tendency rather than a distinct correlation, and is more complicated than expected.

### Expression of Circadian-Regulated Genes and Possible DNA Methylation Regulation in Circadian Rhythm

Using the mRNA sequencing data, a total of 4,937 (11.94% of the total poplar genes) circadian-regulated genes were identified by the LSPR algorithm (Supplementary Table S7), similar to the proportion (8.1–11.2%) of circadian-regulated genes in a previous study of *P. trichocarpa* (Filichkin et al., 2011). GO enrichment analysis of the circadian-regulated genes was performed. Similar to the result for *Arabidopsis* (Harmer et al., 2000), the genes involved in photosynthesis, key metabolic processes, and different cellular components were significantly enriched in poplar circadian-regulated genes (Supplementary Table S8).

The circadian-regulated genes that overlapped with DMRs were identified. Among the 4,937 circadian-regulated genes, 1,895 genes (34.74% of the total) overlapped with 3,516 DMRs obtained between four pairs among five groups (G1 vs. G2, G3 vs. G2, G4 vs. G3, and G5 vs. G4) (Supplementary Table S9). Among the 1,895 circadian-regulated genes, 871 genes were hypermethylated with down-regulated expression levels and 881 genes were hypomethylated with up-regulated expression levels, indicating the possible regulation of DNA methylation on the daily rhythmic expression of these genes.

Among the 53,689 DMRs identified, there were seven DMRs (3 in G2 vs. G1., 3 in G3 vs. G2, 2 in G4 vs. G3, and 2 in G5 vs. G4) located in the CCT domain, a conserved domain of seven genes in CO, COL, and TOC1 (Supplementary Table S10). TOC1 is a core oscillator component of the plant circadian clock, and both CO and COL are regulated by the circadian rhythm (Kim et al., 2003; Zhang et al., 2015). Four of the seven genes expressed in all five groups, and the methylation level in promoter region and/or gene body region, differed between the five groups (Supplementary Figure S24). However, circadian changes in DNA methylation of the promoter region and gene body region, as well as circadian changes in gene expression, were not observed. Meanwhile, about 23 circadian clock-related GO terms and one circadian rhythm pathway were found in DMR overlapping genes within the four comparisons among the five groups (Supplementary Tables S11, S12). Although no rhythmic DNA methylation changes were detected in oscillator component genes of the plant circadian clock, the above results suggest that DNA methylation is at least partially responsible for the transcriptional alterations of these circadian-regulated genes.

## Discussion

DNA methylation is a dynamic regulator of the plant genome during development and environmental adaptions (Mirouze and Paszkowski, 2011; Calarco et al., 2012; Ikeda, 2012; Ma et al., 2015; Xing et al., 2015). Most studies on plant stress responses found obvious changes in DNA methylation patterns after exposure to environmental stimuli (Raj et al., 2011; Yaish et al., 2011; Dowen et al., 2012; Rico et al., 2014; Eichten and Springer, 2015; Ohama et al., 2017), and some studies on plants indicated that DNA methylation changes can occur rapidly. Rapid responses in DNA methylation were observed in *Chorispora bungeana* and *Alternanthera philoxeroides* under cold stress and environmental fluctuations (Gao et al., 2010; Song et al., 2015). In addition to stress, moderate changes in temperature have also been shown to influence the DNA methylation status of plants. For example, the expression of the *Arabidopsis* gene *At3g50770* was correlated with reduced promoter DNA methylation under elevated temperatures (Naydenov et al., 2015). In this study, we found a large amount of DMRs between leaf samples collected at different time points in a day by BS-seq, indicating the daily DNA methylation changes in the plant under unstressed conditions. These DMRs overlapped with nearly one third of the total poplar genes involved in multiple biological processes, various molecular functions, and different cellular components, suggesting the broad involvement of DNA methylation in daily gene expression.

Plants have developed a wide array of mechanisms to adapt to daily environmental conditions, and the regulation of gene expression through both transcriptional regulation and post-transcriptional regulation is very important to their survival. The abundance of plant mRNA in a cell is determined by multiple factors, such as transcriptional factors, chromatin regulation, histone modification of transcriptional regulation, microRNA and mRNA stability, and alternative splicing of post-transcriptional regulation (Floris et al., 2009; Covarrubias and Reyes, 2010; Asensi-Fabado et al., 2017; Laloum et al., 2017; Colinas and Goossens, 2018). Based on the BS-seq and RNA-seq data, we did not detect an overall negative correlation between the methylation and mRNA expression levels in the promoter region, but found an overall positive correlation between the methylation levels in the gene body region and the mRNA expression levels, similar to the previous results in rice and Arabidopsis (Li et al., 2012; Meng et al., 2016; Xu et al., 2018). We suggest that DNA methylation was the main type of post-transcriptional regulation of daily plant gene expression, resulting in the positive correlation between methylation and mRNA expression levels in the gene body region. However, DNA methylation was not the main type of transcriptional regulation in daily plant gene expression, and other types of transcriptional regulation in the promoter region determined the level of gene expression. These combined gene regulation mechanisms resulted in the non-negative correlation between methylation levels in the promoter region and mRNA expression levels.

The circadian clock regulates a large number of genes in plants, and results in rhythmic daily gene expression (Herrero and Davis, 2012). Recent studies in mice and humans have shown that epigenetic mechanisms participated in the circadian clock regulation (Jones et al., 2010; Song and Noh, 2012; Azzi et al., 2014). As a crucial epigenetic mechanism, DNA methylation might be involved in circadian clock regulation in plants, as seen in mammals. In this study, 871 hypermethylated genes were down-regulated in expression levels and 881 hypomethylated genes were up-regulated in expression levels among the 1,895 circadian-regulated genes, indicating the possible regulation of DNA methylation on the daily rhythmic expression of these genes. Meanwhile, GO terms related to the circadian rhythm and circadian clock-related pathway were found in DMR-overlapping genes, and seven DMRs were located in the CCT domain of plant circadian clock genes (TOC1); this indicates involvement of DNA methylation in the poplar circadian clock. Although the expression of four CCT domain genes was detected in all five groups, and their methylation level in the promoter region and/or gene body region differed among the five groups, rhythmic DNA methylation changes and rhythmic gene expression were not observed. Therefore, although DNA methylation regulation was involved in the expression of some circadian regulated genes and a circadian clock gene, it was not the main regulatory mechanism for the circadian system in *Populus*. We suggest that, in addition to chromatin remodeling, which is common to both plants and mammals, other regulatory systems such as histone modification and alternative splicing combined to maintain plasticity and specificity in the plant circadian system (Henriques and Mas, 2013; Barneche et al., 2014; Shen et al., 2016).

The most abundant types of daily DNA methylation change among the three contexts were CHH (63.84%), followed by CHG (28.18%), whereas only approximately 7.98% were in CG. Similar DNA methylation context changes have also been reported in other plants; methylated mCHH was the most abundant type among total significant DMRs, followed by mCG, and mCHG methylation in the date palm (*Phoenix dactylifera* L.) in response to salt stress (Al-Harrasi et al., 2018); the vast majority of DMRs (97%) were in the CHH context, whereas only approximately 3% of DMRs were in CG and CHG during seed development in soybean (*Glycine max* L.) (An et al., 2017). Therefore, methylation in the CG and CHG contexts is more stable in the plant genome than the CHH context in stress response, development regulation, and daily DNA methylation regulation. *MET1*, *CMT3*, and *DRM2*/*CMT2* were the genes involved in the maintenance of DNA methylation in the CG, CHG, and CHH contexts, respectively (Law and Jacobsen, 2010; Stroud et al., 2014; Zhang et al., 2018). To explain the increased CHH context in daily DNA methylation changes, we examined the expression of putative DNA methyltransferases MET1, CMT2, CMT3, DRM2 in our *P. trichocarpa* RNA-seq data. We found that the expression of poplar gene coding for DRM2 was higher than that of the other three DNA methyltransferase genes (Supplementary Figure S25). This finding might partially explain the high proportion of CHH in daily DNA methylation change contexts in our study.

## Materials and Methods

### Plant Material Preparation

*P. trichocarpa* genotype Nisqually-1 was propagated *in vitro* and cultured on hormone-free McCown Woody Plant Medium. Plants were maintained in a growth chamber in a dark room at 25°C under a 16 h photoperiod with a maximum light density of 22,000 LX (PGX-450C, SaiFu Instrument Co., Ltd., Ningbo, China).

Eighteen *in vitro* 6–8-week-old poplar plants of similar growth status were selected for the experiment. These plants were kept in a chamber for 24 h under a 16 h/8 h light/dark cycle and then subjected to continuous illumination for 24 h. Fully extended leaves from two plants (one used for BS-seq and one for mRNA sequencing) were collected at nine time points during the 48 h period, frozen immediately in liquid nitrogen, and then stored at -80°C. The time points used for sample collection and sample grouping in the data analysis are presented in Supplementary Figure S1.

The methylomic and transcriptomic changes in leaves at different time points during the first 24 h under the light/dark cycle reflect diurnal changes, whereas those under the latter 24 h of continuous light reflect inner rhythmic changes. During the latter 24 h, plants were grown under free-running conditions, in which the oscillator drives rhythmicity in the absence of environmental time cues, which is a classic experimental design in circadian research. Samples were placed into one of five groups: G1 [light/dark 0 h (LD0), continuous light 0 h (CL0)], G2 (LD4, CL4), G3 (LD8, CL8), G4 (LD16, CL16), and G5 (CL0, CL24), and the common changes between each pair of groups were considered inner rhythmic methylomic changes. We did not examine the changes under free-running conditions exceeding 24 h, as damping of rhythms often occurs during free-running conditions.

### Bisulfite Sequencing (BS-Seq)

Genomic DNA was isolated from leaves using the standard CTAB method. The library was prepared and sequenced at the Novogene Bioinformatics Institute (Beijing, China) on the Illumina HiSeq 2500 platform.

After low-quality reads were filtered from the raw data using Trimmomatic (version 0.36), clean reads were mapped to the poplar genome^1^ in Bismark (version 0.12.5) using the default parameters (Krueger and Andrews, 2011). The sodium bisulfite non-conversion rate was calculated as the percentage of cytosines sequenced at cytosine reference positions in the lambda genome. Using a sliding window approach, the sum of methylated and unmethylated read counts was calculated using a 3,000 bp window size and a 600 bp step size (Smallwood et al., 2014). The methylation level at each cytosine site shows the fraction of methylated cytosines, defined as ML = mC/(mC + umC), where ML is the methylation level, mC, and umC are methylated and unmethylated cytosine, respectively. The methylation level was further corrected using the bisulfite non-conversion rate according to a previous study (Lister et al., 2013). Methylation density was calculated by dividing the number of methylated cytosines by the total number of cytosines within a region.

### DMC, DMR, and Differentially Methylated Gene Identification

The DMCs were defined by Fisher’s exact test using FDR (false discovery rate) multiple test correction. DMCs with a corrected *p*-value < 0.05 and a difference in the methylation level of >0.2 between two groups were considered candidate DMCs. The regions in the genome with a methylation level difference >0.1, at least three DMCs, and a distance between adjacent DMCs <300 bp were considered DMRs. The genes in which the gene body region or promoter region (2 kb upstream of the TSS) overlapped with DMRs were considered differentially methylated genes.

### GO Enrichment Analysis

GO enrichment analysis was implemented in the agriGO v. 2.0 software (Tian et al., 2017). GO terms with FDA values <0.05 were considered significantly enriched.

### RNA Sequencing (RNA-Seq) and Data Analysis

Total RNA was isolated from leaves using the RNA plant Plus Reagent (Tiangen Biotech, Beijing, China). Libraries were constructed using the NEBNext Ultra RNA Library Prep Kit for Illumina (NEB, Ipswich, MA, United States) following the manufacturer’s recommendations. The library preparations were sequenced on the Illumina HiSeq 4000 platform (paired-end 125/150 bp reads).

High-quality clean reads were mapped to the *P. trichocarpa* genome using TopHat version 2.0.12. Transcript expression levels were estimated using the FPKM (fragments per kilobase per million fragments mapped) method in HTSeq version 0.6.1.

### Rhythmic Gene Identification

Rhythmic genes were identified using the LSPR algorithm with default settings (Yang et al., 2011).

### Validation BS-Seq Data by Traditional Bisulfate PCR (BS-PCR) and Sanger Sequencing

The DNA Bisulfite Conversion Kit (Tiangen, China) were used for Bisulfite conversions. Bisulfite-modified PCR primers were designed using the online program BiSearch^2^ (Supplementary Table S13). PCR was performed using Ex Taq Hot Start Polymerase (Takara, Japan) in 50-μl reaction volumes containing 200 ng template DNA and 50 pmol of primers. The PCR amplification conditions were: 5 min at 95°C, followed by 50 cycles of 1 min at 95°C, 1 min at 56°C, and 20 s at 72°C, and further elongation at 72°C for 5 min using an ABI Veriti 96-Well Thermal Cycler (Applied Biosystems). The PCR products were gel separated, purified with an AxyPrep DNA gel extraction kit (Axygen, United States). Purified amplicons were cloned into the pMD19-T vector (Takara, Japan) and sequenced. The methylation level was calculated by dividing the number of non-converted (methylated) cytosines by the total number of cytosines in the amplified fragment.

### First-Strand cDNA and Quantitative Real-Time PCR (qPCR) Analysis

The cDNA was synthesized using PrimeScript 1st Strand cDNA Synthesis Kit (Takara, Japan) according to the protocol provided by the manufacturer. The qPCR was conducted on a LightCycler 480 System platform (Roche, Switzerland). Each 20 μl PCR system contained 1 μl of first-strand cDNA, 200 nM of primers and 1× SYBR PCR mixture (TaKaRa, Japan). The amplification conditions were: 10 s at 95°C, followed by 45 cycles of 10 s at 95°C, 10 s at 60°C, and 10 s at 72°C. We performed three to four replicates for each sample. Relative quantification values were calculated using the 2^-ΔΔCT^ method (Livak and Schmittgen, 2001). Amplification lengths and primers are listed in Supplementary Table S14.

## Data Availability

The data from this study are available upon request.

## Author Contributions

BZ and XS designed the experiments. LL prepared the plant samples. LL, YC, and JL conducted the experiments and analyzed the data. LL, YC, and BZ wrote the manuscript, with the help of JL, XW, QL, WZ, and XS.

## Conflict of Interest Statement

The authors declare that the research was conducted in the absence of any commercial or financial relationships that could be construed as a potential conflict of interest.

## Footnotes

1 ftp://ftp.ensemblgenomes.org/pub/release-23/plants/fasta/populus_trichocarpa/dna/

2 http://bisearch.enzim.hu/?m=search
