# Supplementary material for: Global Methylomic and Transcriptomic Analyses Reveal the Broad Participation of DNA Methylation in Daily Gene Expression Regulation of Populus trichocarpa
Source: Front Plant Sci. 2019 Feb 28;10:243. doi: 10.3389/fpls.2019.00243 (PMC6403135; doi:10.3389/fpls.2019.00243)
Supplement: Supplementary file 1 [file Data_Sheet_1.zip › Data Sheet 1/Supplementary Figures.DOCX]

**Global methylomic and transcriptomic analyses reveal the broad participation of DNA methylation in daily gene expression regulation of *Populus trichocarpa***

**Li-Xiong Liang^1,^ ^2^ *, Ying-Ying Chang^1,2^* , Jun-Qian Lu^1,2^, Xiao-Juan Wu^1,2^, Qi Liu^1,2^, Wei-xi Zhang^1,2^, Xiao-Hua Su^1,2^, Bing-Yu Zhang^1,2^**

^1^ State Key Laboratory of Tree Genetics and Breeding, Research Institute of Forestry, Chinese Academy of Forestry, Beijing, China

^2^ Key Laboratory of Tree Breeding and Cultivation of State Forestry Administration, Research Institute of Forestry, Chinese Academy of Forestry, Beijing, China

* These authors contributed equally to the work.

Correspondence and requests for materials should be addressed to X.H.S and B.Y.Z. (email: [**suxh@caf.ac.cn**](mailto:suxh@caf.ac.cn)**,** [byzhang@caf.ac.cn](mailto:byzhang@caf.ac.cn))

Figure S1∣ Time points for sample collection. Samples were placed into one of five groups: G1 (light/dark 0 h (LD0), continuous light 0 h (CL0)), G2 (LD4, CL4), G3 (LD8, CL8), G4 (LD16, CL16), and G5 (CL0, CL24), to detect inner rhythmic methylomic and transcriptomic changes.


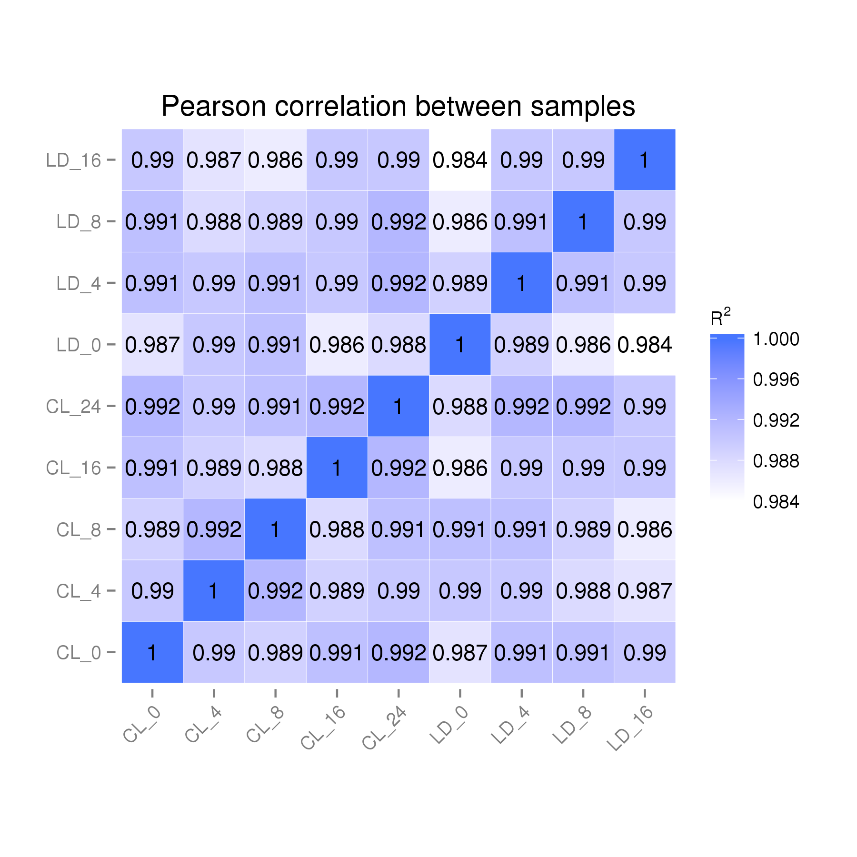


Figure S2∣Pearson correlation between samples.

Figure S3∣ Fraction of 5-methylcytosines identified in each context (CG, CHG and CHH) for poplar, tomato and Arabidopsis. Fraction of 5-methylcytosines in poplars are similar to tomato fruit, but in contrast to *Arabidopsis* leaves.


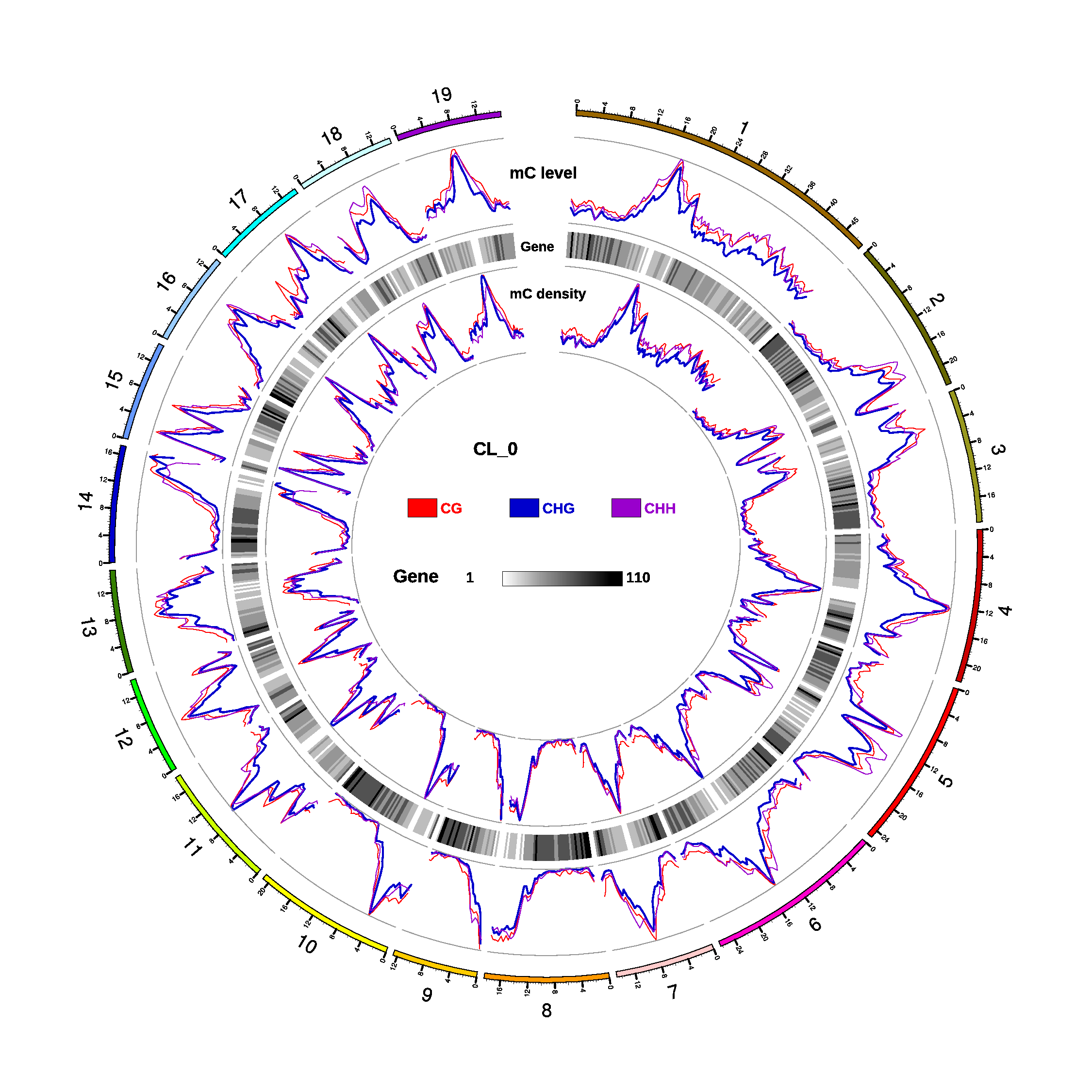


Figure S4∣ Chromosome distribution of methylation density and level in sample CL_0. In each chromosome, the parts with high methylation density and high level of methylation were sparse in genes. Chromosome distribution of mC density and methylation level in all samples were similar, and not present in here.


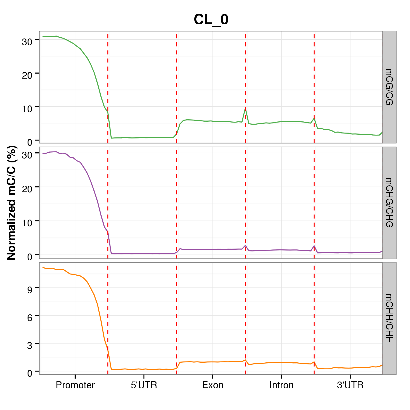

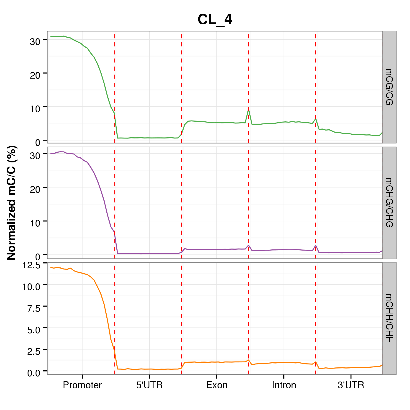

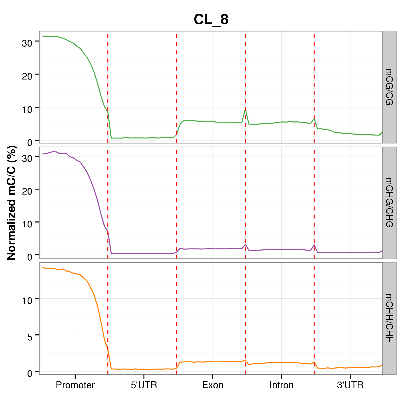

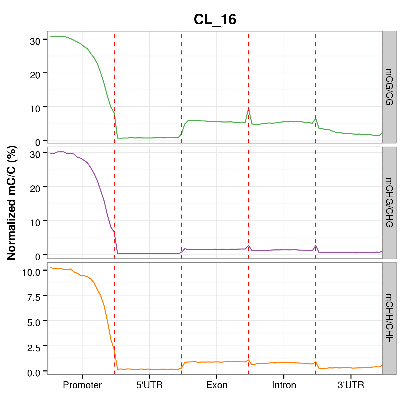

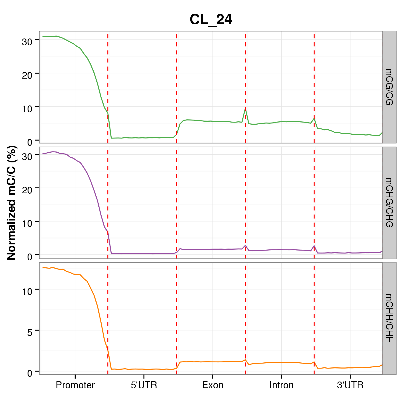

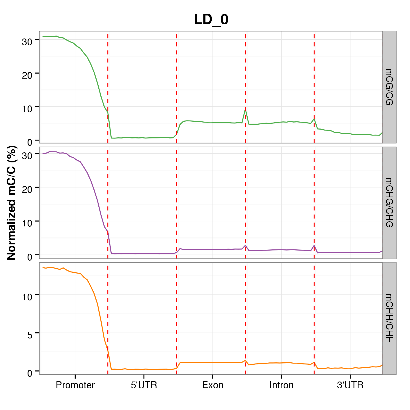

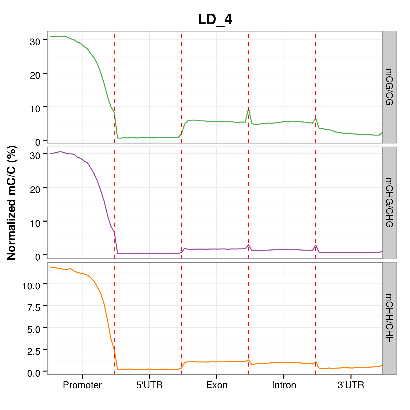

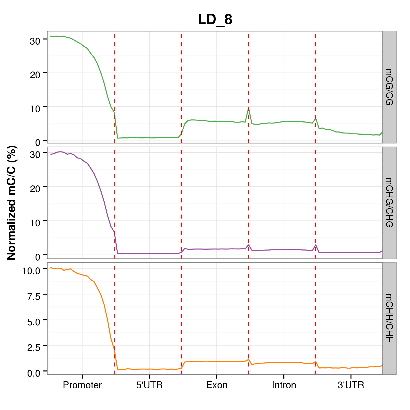

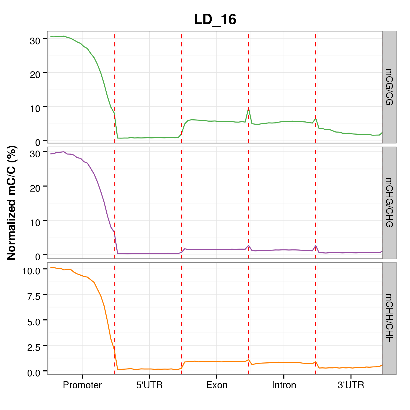


Figure S5∣ Methylation density of CG, CHG and CHH in gene body of sample LD_8. Methylation density of all the three contexts were much higher in promoter regions than in the exons, introns and UTRs. Genomic element regions were divided into 20 bins and average methylation density in CG, CHG and CHH context was calculated. Promoter: 2 kb upstream of the TSS. Other samples were not presented here.


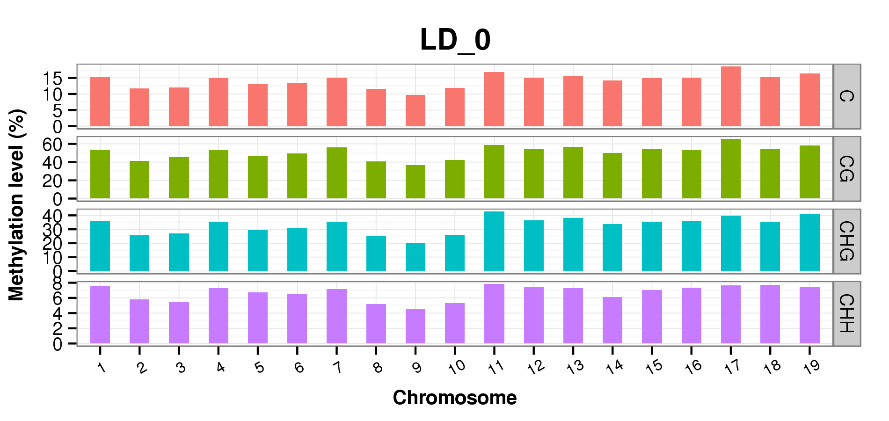


Figure S6∣Average ^m^C level per chromosome of sample LD_0. The average methylation level in each chromosome differed, with the lowest in chromosome 9 and the highest in chromosome 17. Other samples were not presented here.


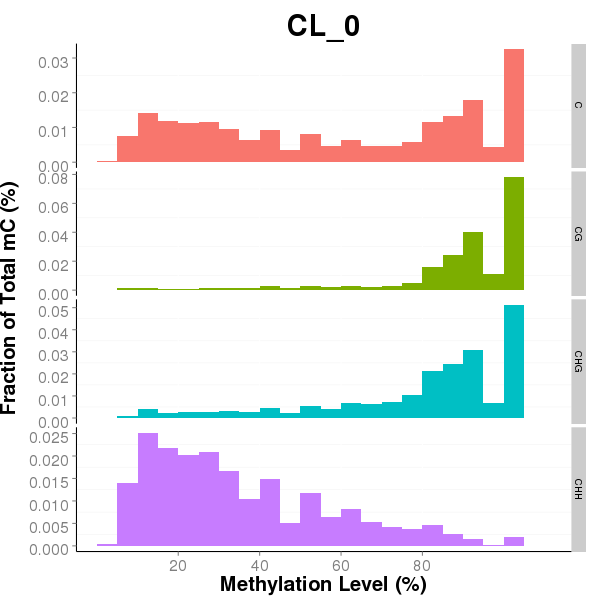


Figure S7∣Methylation level distribution of CG, CHG and CHH context in sample CL_0, with the highest levels at CG, medium levels at CHG, and the lowest levels at CHH sites Other samples were not presented here.


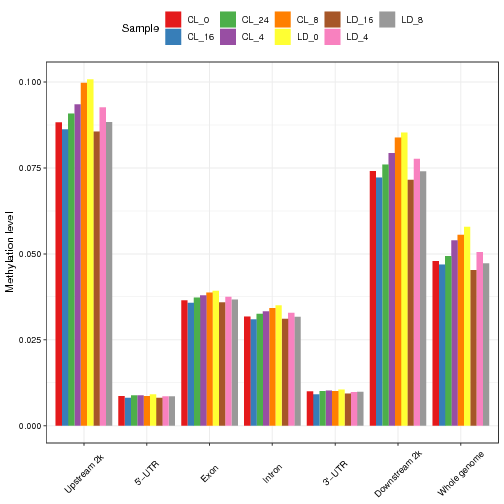


Figure S8∣Methylation levels of different genic regions in all samples. Methylation levels were high in upstream and downstream of the transcribed region, medium in exons and introns, low in untranslated regions (5’-UTR and 3’-UTR).

Figure S9∣Methylation levels of different TEs types. The hAT and CMC-EnSpm transposons tended to be more heavily methylated than other TEs.

Figure S10∣Validation of the methylation level of a 294bp exon region (chr12_9852031-9852327) of gene POPTR_0012s08770s in three randomly selected samples (CL0, CL4 and CL8), using the traditional bisulfite sequencing method. The methylation level was shown at the y axis and the genomic location was shown at the x axis.

Figure S11∣Validation of the methylation level of a 269bp promoter region region (chr15_1573578-1573846) of gene POPTR_0015s02230 in three randomly selected samples (LD4, CL0 and CL8) using the traditional bisulfite sequencing method. The methylation level was shown at the y axis and the genomic location was shown at the x axis.

Figure S12∣DMRs (differentially methylated regions) identified between four pairs of five groups (G1 vs. G2, G3 vs. G2, G4 vs. G3, and G5 vs. G4).

Figure S13. DMRs distribution in different genic regions. Most DMRs located in promoter regions, indicating their potential regulation on downstream gene expression.

Figure S14∣The CG, CHG, and CHH contexts of DMCs. The CG, CHG, and CHH contexts of the DMCs differed, with the highest proportion located in CHH, followed by CHG, and the lowest in CG.

Figure S15∣DMCs distribution in different genic regions. Most DMCs (70.59%) located in the in the promoter regions, 14.19% in exon, 13.91% in intron and 1.31% in UTRs.


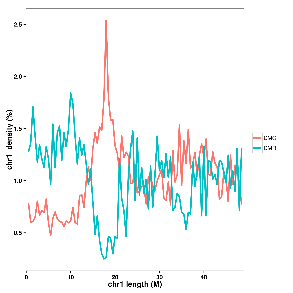

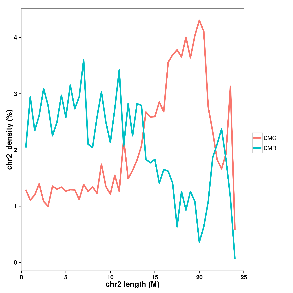

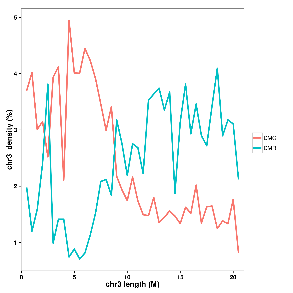

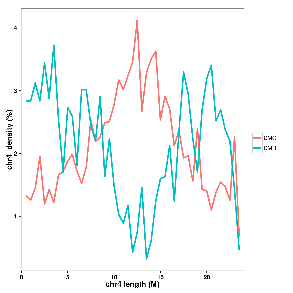

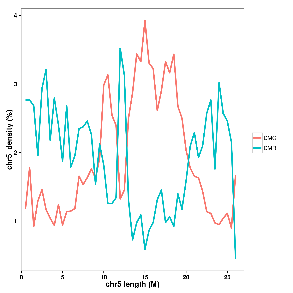

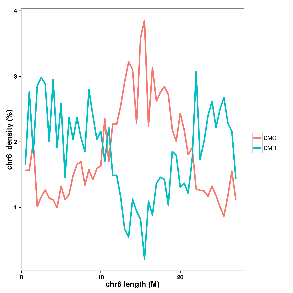

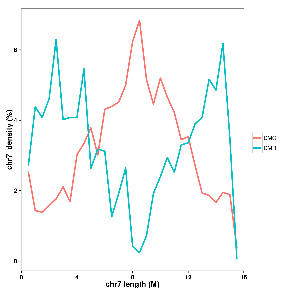

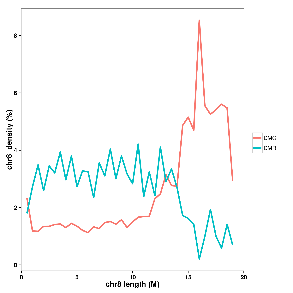

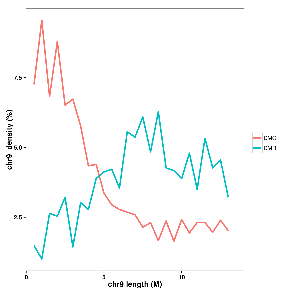

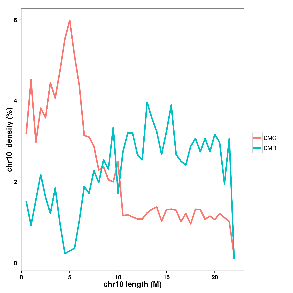

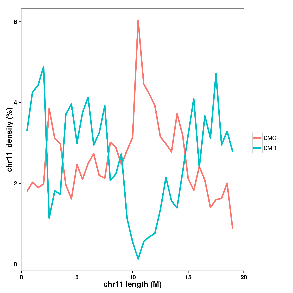

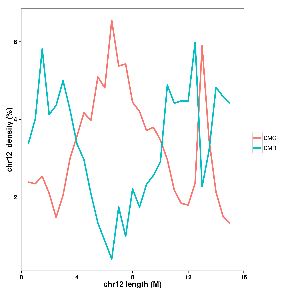

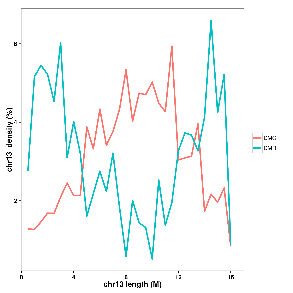

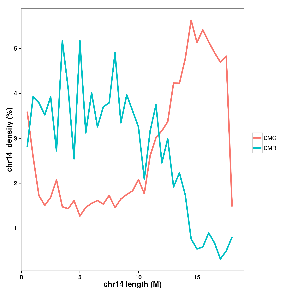

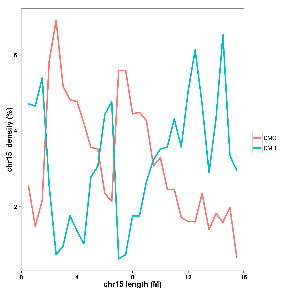

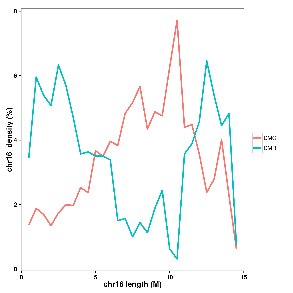

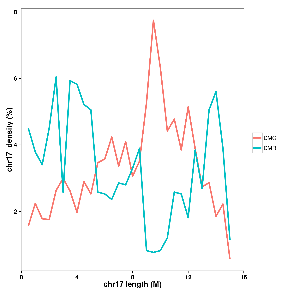

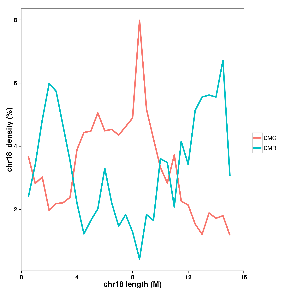

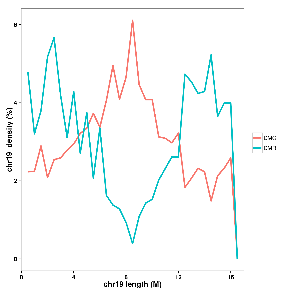


Figure S16∣An uneven distribution of DMRs and DMCs in all 19 chromosomes (bin size, 0.5M).


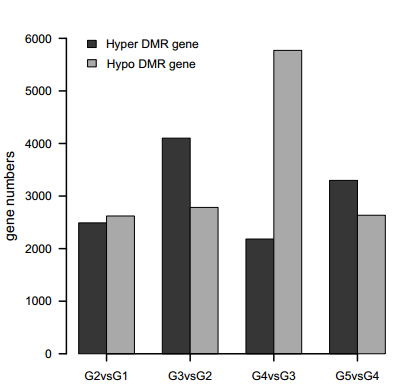


Figure S17∣The genes overlapped with hyper DMRs and hypo DMRs in the four comparisons (G1 vs G2, G3 vs G2, G4 vs G3 and G5 vs G4).


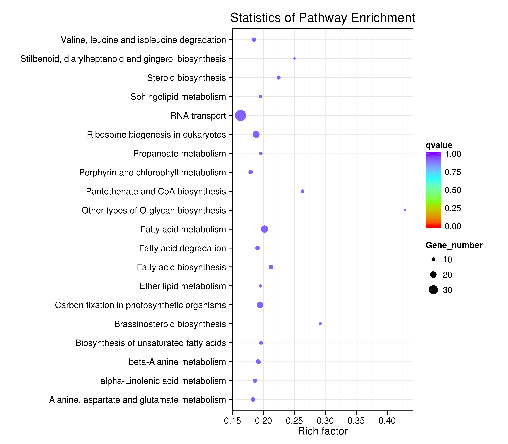

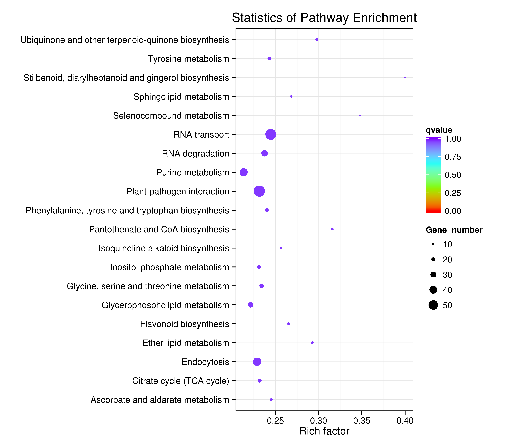

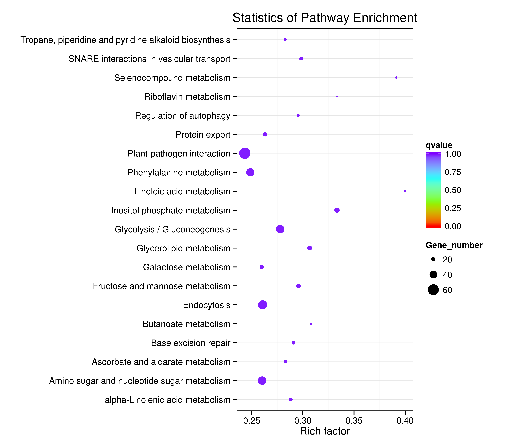

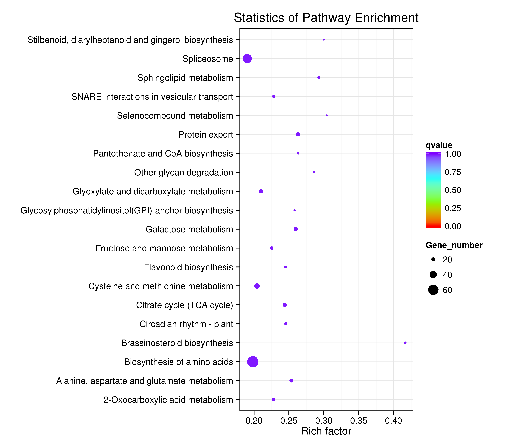


Figure S18∣KEGG pathway Scatterplot of DMR overlapping genes. There were not significant pathway were found in the analysis.


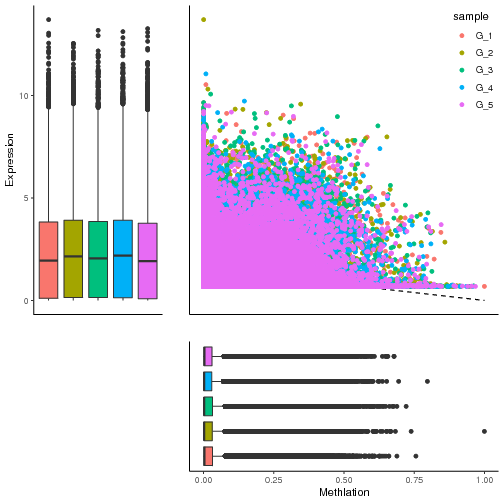

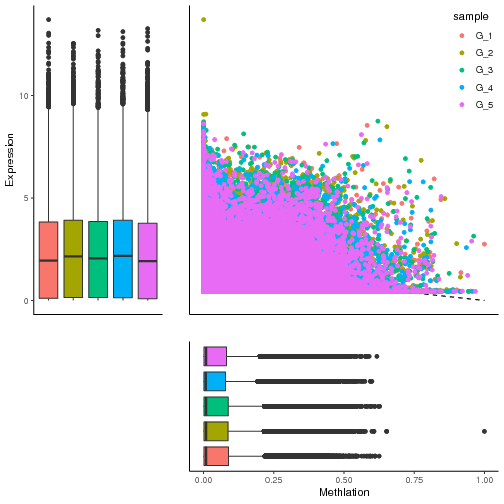


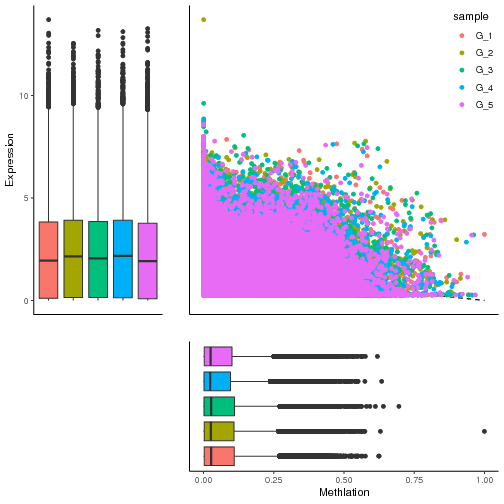

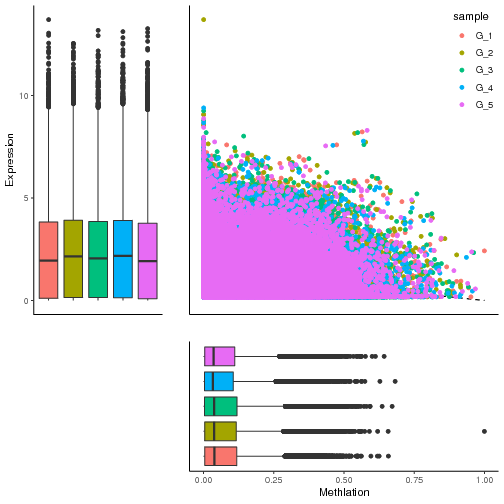


Figure S19∣Scatterplot of DNA methylation (x-axis) and expression (y-axis) in 0.5kb to 2.0kb upstream the transcriptional start site (TSS) in five groups (G1, G2, G3, G4 and G5). (A) 0.5kb. (B) 1.0kb. (C) 1.5kb. (D) 2.0kb. Negative correlation between the methylation levels and the mRNA expression levels was not observed.


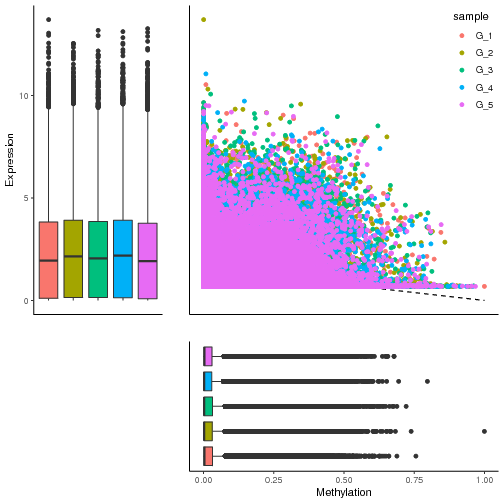

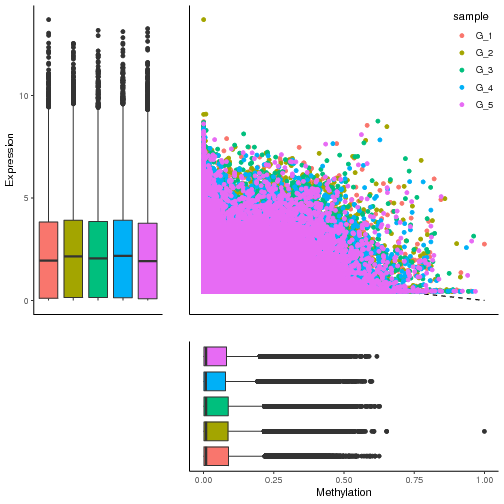


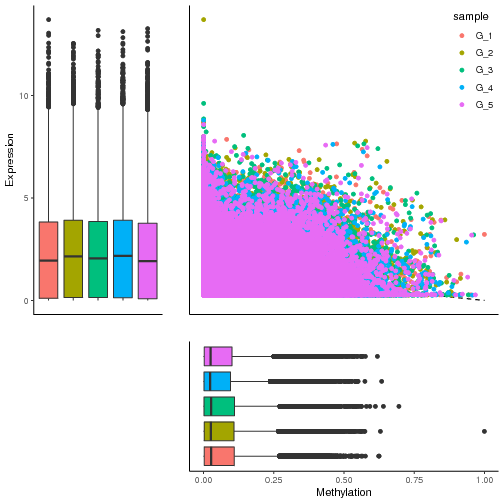

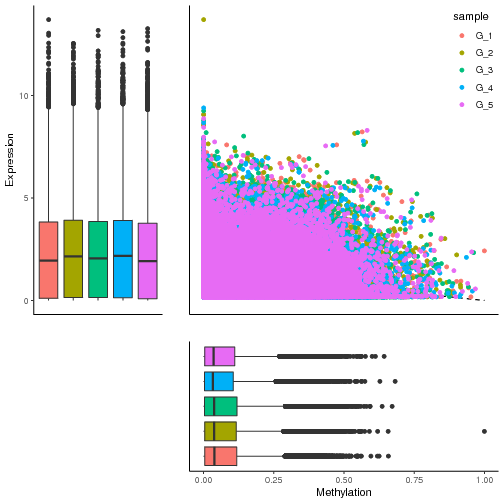


Figure S20∣Scatterplot of DNA methylation (x-axis) and expression (y-axis) in 0.5kb to 2.0kb downstream the transcriptional end site (TES) in five groups (G1, G2, G3, G4 and G5). (A) 0.5kb. (B) 1.0kb. (C) 1.5kb. (D) 2.0kb. Slightly negative correlation between the methylation levels and the mRNA expression levels was observed in shorter downstream of the transcribed region of genes.


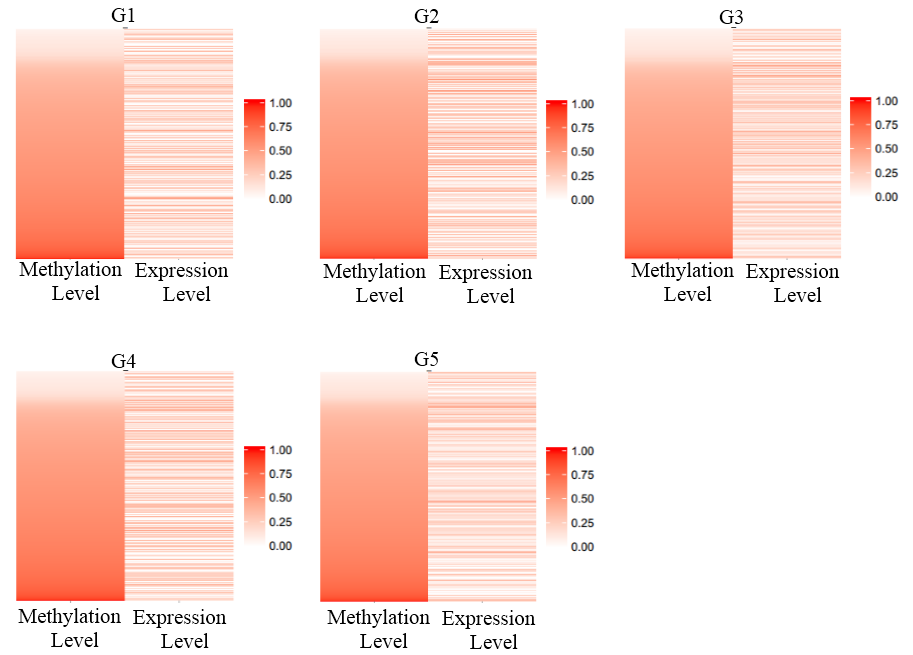


Figure S21∣The methylation levels in the promoter regions of genes overlapping with DMRs in each group and their corresponding expression levels. A negative correlation between the methylation level in the promoter region and the mRNA expression level of that genes were observed for certain genes.

Figure S22∣The expression results of the six circadian regulated genes got from the transcriptome data and quantitative -real-time PCR(qPCR). The expression tendency of the six genes was generally consistent with the RNA-seq results.

Figure S23∣Expression validation of six circadian regulated genes and their methylation level in promoter and gene body region.


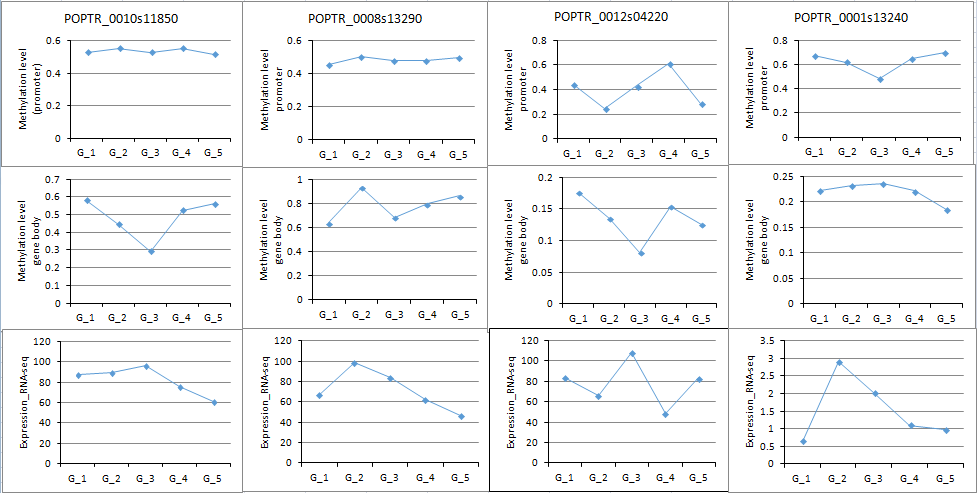


Figure S24∣Transcriptomic expression of the four CCT domain genes in five groups and their methylation level in promoter region and/or gene body.

Figure S25∣The expression of putative DNA methyltransferases MET1, CMT2, CMT3, DRM2 of *P. trichocarpa* from RNA-seq data.
