# Supplementary material for: Global Methylomic and Transcriptomic Analyses Reveal the Broad Participation of DNA Methylation in Daily Gene Expression Regulation of Populus trichocarpa
Source: Front Plant Sci. 2019 Feb 28;10:243. doi: 10.3389/fpls.2019.00243 (PMC6403135; doi:10.3389/fpls.2019.00243)
Supplement: Supplementary file 1 [file Data_Sheet_1.zip › Data Sheet 1/Supplementary Tables.DOCX]

**Global methylomic and transcriptomic analyses reveal the broad participation of DNA methylation in daily gene expression regulation of *Populus trichocarpa***

**Li-Xiong Liang^1,^ ^2^ *, Ying-Ying Chang^1,2^* , Jun-Qian Lu^1,2^, Xiao-Juan Wu^1,2^, Qi Liu^1,2^, Wei-xi Zhang^1,2^, Xiao-Hua Su^1,2^, Bing-Yu Zhang^1,2^**

^1^ State Key Laboratory of Tree Genetics and Breeding, Research Institute of Forestry, Chinese Academy of Forestry, Beijing, China

^2^ Key Laboratory of Tree Breeding and Cultivation of State Forestry Administration, Research Institute of Forestry, Chinese Academy of Forestry, Beijing, China

* These authors contributed equally to the work.

Correspondence and requests for materials should be addressed to X.H.S and B.Y.Z. (email: [**suxh@caf.ac.cn**](mailto:suxh@caf.ac.cn)**,** [byzhang@caf.ac.cn](mailto:byzhang@caf.ac.cn))

Table S1. Summary of sequencing results of BS-seq libraries, both strands combined.

| Sample | Clean Reads | Clean Bases | Error Rate | Q20 | Q30 | GC Content | BS Conversion Rate (%) |
| --- | --- | --- | --- | --- | --- | --- | --- |
|  |  | (G) | (%) | (%) | (%) | (%) |  |
| LD_0 | 140,466,810 | 17.56 | 0.02 | 96.62 | 92.1 | 19.04 | 99.8 |
| LD_4 | 148,933,328 | 18.62 | 0.02 | 96.66 | 92.3 | 19.14 | 99.84 |
| LD_8 | 138,094,666 | 17.26 | 0.02 | 96.3 | 91.5 | 19.2 | 99.82 |
| LD_16 | 149,583,400 | 18.7 | 0.02 | 96.93 | 92.4 | 19.17 | 99.83 |
| CL_0 | 155,184,186 | 19.4 | 0.02 | 96.52 | 91.9 | 19.09 | 99.8 |
| CL_4 | 156,332,784 | 19.54 | 0.02 | 95.81 | 89.8 | 18.64 | 99.79 |
| CL_8 | 203,699,526 | 25.46 | 0.02 | 96.16 | 90.6 | 19.34 | 99.85 |
| CL_16 | 153,047,416 | 19.13 | 0.02 | 96.57 | 91.9 | 18.91 | 99.83 |
| CL_24 | 202,203,970 | 25.28 | 0.02 | 95.8 | 90.1 | 19.18 | 99.77 |

Table S2.Mapping rate and cytocine of bisulfite sequencing.

| Sample | Total reads | Mapped reads | Mapping rate (%) | Duplication rate (%) | 1x C coverage(%) | 5x C coverage(%) |
| --- | --- | --- | --- | --- | --- | --- |
| LD_0 | 70,233,405 | 49,698,380 | 70.76 | 9.19 | 95.69 | 93.41 |
| LD_4 | 74,466,664 | 52,907,879 | 71.05 | 10.78 | 95.64 | 93.76 |
| LD_8 | 69,047,333 | 45,884,107 | 66.45 | 10.12 | 95.58 | 93.42 |
| LD_16 | 74,791,700 | 50,325,536 | 67.29 | 16.27 | 95.73 | 93.67 |
| CL_0 | 77,592,093 | 53,470,023 | 68.91 | 10.67 | 95.7 | 93.92 |
| CL_4 | 78,166,392 | 51,720,030 | 66.17 | 10.4 | 95.75 | 93.46 |
| CL_8 | 101,849,763 | 69,890,479 | 68.62 | 17.06 | 95.68 | 94.07 |
| CL_16 | 76,523,708 | 51,947,600 | 67.88 | 16.05 | 95.71 | 93.64 |
| CL_24 | 101,101,985 | 67,596,148 | 66.86 | 15.87 | 95.76 | 94.25 |

Table S3. Methylation levels in each sequence context of the 9 samples.

| Sample | mC(%) | mCpG(%) | mCHG(%) | mCHH(%) |
| --- | --- | --- | --- | --- |
| LD_0 | 17.40 | 42.53 | 29.01 | 12.38 |
| LD_4 | 17.29 | 44.33 | 30.15 | 11.82 |
| LD_8 | 15.65 | 42.98 | 28.92 | 10.07 |
| LD_16 | 15.63 | 42.73 | 28.51 | 10.16 |
| CL_0 | 16.80 | 44.74 | 30.15 | 11.14 |
| CL_4 | 16.64 | 42.66 | 29.22 | 11.34 |
| CL_8 | 19.50 | 46.38 | 31.98 | 14.13 |
| CL_16 | 15.63 | 42.88 | 28.83 | 10.08 |
| CL_24 | 18.64 | 46.65 | 31.81 | 13.00 |
| average | 17.02 | 43.99 | 29.84 | 11.57 |

Table S4. The ratio of hypo- to hyper-methylated DMRs in four pairs of five groups (G2 vs. G1, G3 vs. G2, G4 vs. G3, and G5 vs. G4).

|  | hypo DMRs | hyper DMRs | hypo/hyper DMRs |
| --- | --- | --- | --- |
| G2 vs G1 | 5,869 | 5,437 | 1.08 |
| G3 vs G2 | 5,615 | 8,109 | 0.69 |
| G4 vs G3 | 12,206 | 4,430 | 2.76 |
| G5 vs G4 | 5,890 | 7,685 | 0.77 |
| total | 29,580 | 25,661 | 1.15 |

Table S6. Summary statistics of RNA-seq.

| Sample | Total reads | mapped reads | mapped rate(%) | Uniquely mapped | Uniquely mapped rate (%) | Exon  (%) | Inter-  genic  (%) | Intron  (%) |
| --- | --- | --- | --- | --- | --- | --- | --- | --- |
| LD_0 | 46341086 | 41559048 | 89.68 | 40821425 | 88.09 | 95.20 | 3.10 | 1.70 |
| LD_4 | 43581062 | 39251582 | 90.07 | 38415596 | 88.15 | 94.50 | 3.50 | 1.90 |
| LD_8 | 51255258 | 45819843 | 89.40 | 44915358 | 87.63 | 95.30 | 3.00 | 1.70 |
| LD_16 | 44912596 | 40660257 | 90.53 | 39935273 | 88.92 | 95.30 | 2.80 | 1.90 |
| CL_0 | 42336522 | 38501703 | 90.94 | 37884928 | 89.49 | 96.10 | 2.20 | 1.60 |
| CL_4 | 47242282 | 43012823 | 91.05 | 42195604 | 89.32 | 95.60 | 2.50 | 1.90 |
| CL_8 | 44301094 | 40122255 | 90.57 | 39279606 | 88.67 | 96.30 | 2.30 | 1.50 |
| CL_16 | 41084384 | 37400846 | 91.03 | 36716828 | 89.37 | 95.30 | 2.70 | 2.00 |
| CL_24 | 45001092 | 40840711 | 90.75 | 40166091 | 89.26 | 95.80 | 2.40 | 1.80 |

Table S8. Significantly enriched GO terms of the circadian-regulated genes in *P. trichocarpa.*

| GO term | Ontology | Description | Number in input list | Number in BG/Ref | p-value | FDR |
| --- | --- | --- | --- | --- | --- | --- |
| GO:0006412 | P | translation | 115 | 611 | 1.90E-08 | 3.50E-05 |
| GO:0019684 | P | photosynthesis, light reaction | 21 | 41 | 1.00E-07 | 6.30E-05 |
| GO:0006091 | P | generation of precursor metabolites and energy | 45 | 164 | 1.00E-07 | 6.30E-05 |
| GO:0009765 | P | photosynthesis, light harvesting | 20 | 39 | 2.00E-07 | 9.40E-05 |
| GO:0015979 | P | photosynthesis | 29 | 88 | 8.90E-07 | 0.00028 |
| GO:0044237 | P | cellular metabolic process | 815 | 6668 | 9.10E-07 | 0.00028 |
| GO:0008152 | P | metabolic process | 1114 | 9587 | 2.20E-05 | 0.006 |
| GO:0009987 | P | cellular process | 1001 | 8559 | 2.90E-05 | 0.0069 |
| GO:0003735 | F | structural constituent of ribosome | 89 | 434 | 2.80E-08 | 3.50E-05 |
| GO:0005198 | F | structural molecule activity | 95 | 507 | 3.70E-07 | 0.00023 |
| GO:0030529 | C | ribonucleoprotein complex | 97 | 476 | 8.80E-09 | 2.80E-06 |
| GO:0005840 | C | ribosome | 89 | 434 | 2.80E-08 | 4.30E-06 |
| GO:0043232 | C | intracellular non-membrane-bounded organelle | 110 | 600 | 1.20E-07 | 9.50E-06 |
| GO:0043228 | C | non-membrane-bounded organelle | 110 | 600 | 1.20E-07 | 9.50E-06 |
| GO:0032991 | C | macromolecular complex | 176 | 1145 | 1.30E-06 | 8.30E-05 |
| GO:0005622 | C | intracellular | 433 | 3314 | 2.10E-06 | 0.0001 |
| GO:0005737 | C | cytoplasm | 177 | 1165 | 2.30E-06 | 0.0001 |
| GO:0044444 | C | cytoplasmic part | 143 | 938 | 1.80E-05 | 0.0007 |
| GO:0044424 | C | intracellular part | 328 | 2492 | 2.30E-05 | 0.0008 |
| GO:0043229 | C | intracellular organelle | 254 | 1910 | 0.00011 | 0.0031 |
| GO:0043226 | C | organelle | 254 | 1910 | 0.00011 | 0.0031 |
| GO:0044464 | C | cell part | 673 | 5684 | 0.00026 | 0.0062 |
| GO:0005623 | C | cell | 673 | 5684 | 0.00026 | 0.0062 |
| GO:0005739 | C | mitochondrion | 26 | 124 | 0.0016 | 0.036 |

Table S13. Primers used in BS-PCR.

| Chromosome location | Amplification Length | Primer sequence | Genic Region |
| --- | --- | --- | --- |
| Chr12  9852031-9852327 | 335 bp | 5’-GTTGATATAATYTTGYTTGYGTTTT-3’ | Promoter |
|  |  | 5’- CAAACTRAARATRCCCACCTCTATC-3’ |  |
| Chr15 1573578-1573846 | 329 bp | 5’- GATTTGYTTTTTTGGAGGTYAAATT-3’ | Promoter |
|  |  | 5’- CATRACAATATRAACTTARAATACA-3’ |  |

Table S14. Primers used in the qPCR.

| Genes ID | Amplification Length | Primer sequence |
| --- | --- | --- |
| POPTR_0012s08770 | 186 bp | 5’-TGGTGTTTTAGCCTCTGCTC -3’ |
|  |  | 5’-TTGACTCCTTCAGACGAAGC -3’ |
| POPTR_0006s06900 | 230 bp | 5’-ACCATTCCGGAACCTTCTTC -3’ |
|  |  | 5’-TCCCTGACATTCTGCAACAG -3’ |
| POPTR_0007s05030 | 280 bp | 5’-AAGCCAATATGAGGCTGGTG -3’ |
|  |  | 5’-TCTGCAGATGGTCGTTTCAG -3’ |
| POPTR_0009s07950 | 201 bp | 5’-GCTGATGGGTGTTTCATGTG -3’ |
|  |  | 5’-TCAGAGCCAACACGATTCTG -3’ |
| POPTR_0008s10480 | 227bp | 5’-TGCTGGTGCTAAAAGTGGTG -3’ |
|  |  | 5’-CCCGAACTTTTCCACTCTTG -3’ |
| POPTR_0015s02230 253bp | | 5’-GCGGAAGAGCTCATATTTGG -3’ |
|  |  | 5’-GTGCATGTAATGCCATCTCG -3’ |
